# Destroyed lung due to sustained inflammation after chemoradiotherapy followed by durvalumab

**DOI:** 10.1002/rcr2.580

**Published:** 2020-05-10

**Authors:** Kageaki Taima, Hisashi Tanaka, Masamichi Itoga, Yoshiko Ishioka, Akira Kurose, Sadatomo Tasaka

**Affiliations:** ^1^ Department of Respiratory Medicine Hirosaki University Graduate School of Medicine Hirosaki Japan; ^2^ Department of Anatomic Pathology Hirosaki University Graduate School of Medicine Hirosaki Japan

**Keywords:** *Aspergillus fumigatus*, chemoradiotherapy, destroyed lung, durvalumab, squamous cell carcinoma

## Abstract

A 68‐year‐old male patient with squamous cell carcinoma (cT4N2M0) of the left upper lobe received chemoradiotherapy followed by durvalumab, an immune checkpoint inhibitor. The tumour responded well to the therapy, but an infiltrative shadow appeared in the left upper lobe, which was outside the radiation field. Despite treatment with corticosteroid and antibiotics, the development of a cavitary lesion was noted. As *Aspergillus fumigatus* was isolated from the bronchoscopy specimen, antifungal agents were also administered, but the cavitary lesion further developed. Because his general condition worsened and the entire left lung was destroyed, the patient underwent a left pneumonectomy and recovered without recurrence. The pathology of the removed lung revealed a scarred nodule with granulation tissue around and a cavernous lesion having a necrotic substance inside. We considered that durvalumab might further accelerate the inflammatory response, which had been introduced by fungal infection, leading to uncontrollable inflammation of the lung.

## Graphical abstract

This report has presented a case of destroyed lung in a patient with non‐small cell lung cancer (NSCLC) who received chemoradiotherapy followed by durvalumab. Because of the sustained inflammation and abolished function of the left lung, left pneumonectomy was required. We considered that durvalumab might further accelerate the inflammatory response, which had been introduced by fungal infection, leading to uncontrollable inflammation of the lung.

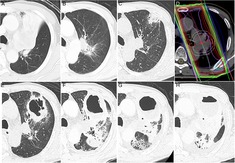

## Introduction

Durvalumab is a therapeutic monoclonal antibody that blocks programmed death ligand 1 (PD‐L1), resulting in T‐cell activation and an antitumour response [1]. In many countries, durvalumab has been approved for patients with unresectable stage III non‐small cell lung cancer (NSCLC) when the disease has not progressed following platinum‐based chemoradiotherapy (CRT). The approval for durvalumab followed the findings from the PACIFIC phase III clinical trial, which showed that adding durvalumab after CRT for stage III NSCLC resulted in significantly improved progression‐free survival (PFS) [1]. The use of durvalumab and other immune checkpoint inhibitors is associated with unique adverse events called immune‐related adverse events (irAEs), which are caused by the disruption of homeostasis of the immune system. On the other hand, little is known about the relationship between infectious disease and cancer immunotherapy.

We experienced a case of destroyed lung due to sustained inflammation in a patient with NSCLC who received CRT followed by durvalumab. Although left pneumonectomy was required because of the abolished function of the left lung, the patient recovered and has been surviving without recurrence of lung cancer.

## Case Report

**Figure 1:**
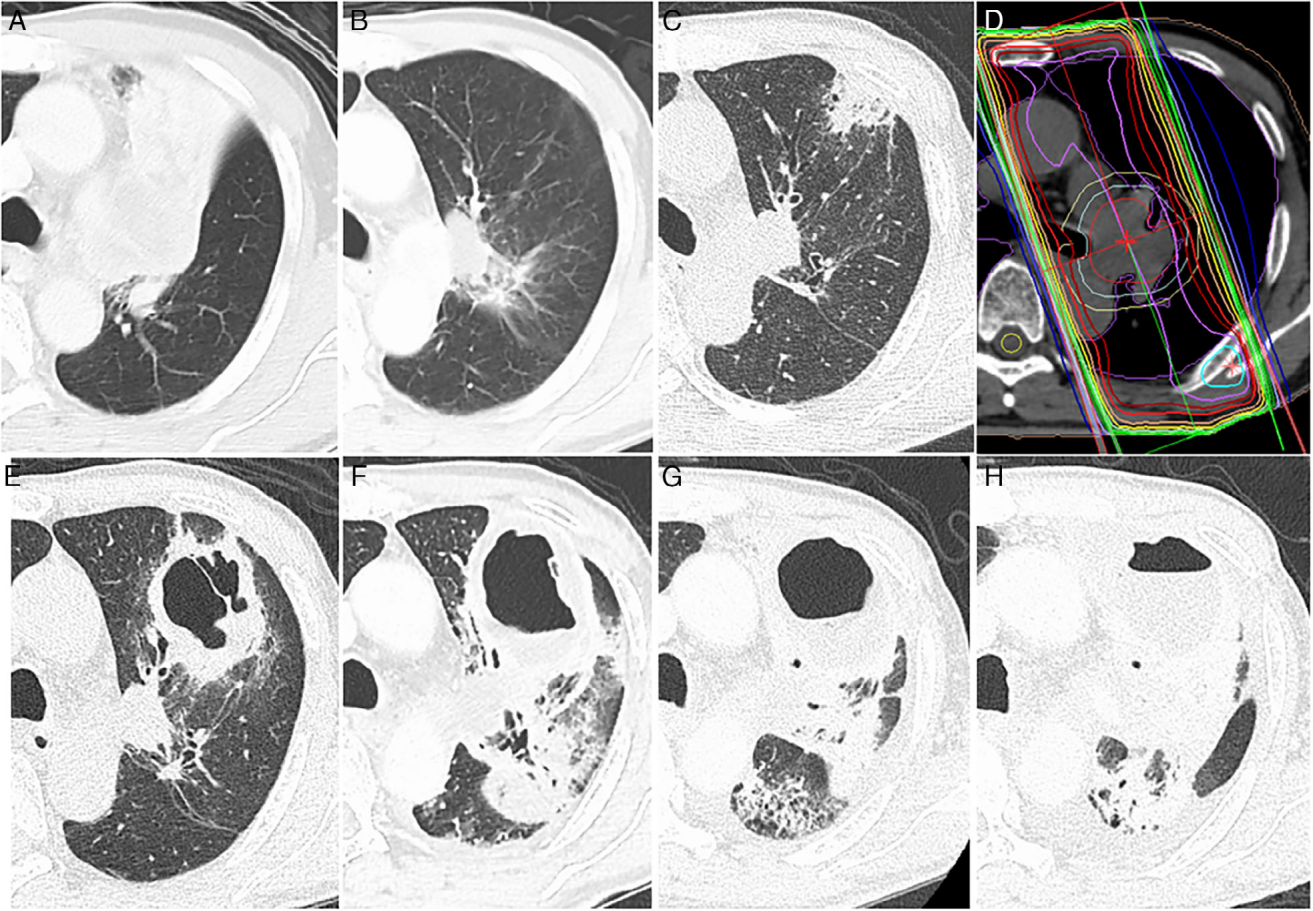
Computed tomography (CT) scan taken at diagnosis of lung cancer showing a hilar tumour causing atelectasis of the left upper lobe (A). CT scan taken after completion of chemoradiotherapy revealing marked decrease in the primary lesion as well as resolution of the atelectasis (B). CT scan on readmission showing lung infiltrate in the left upper lobe (C). CT imaging for radiotherapy planning indicating that the lung infiltrate was outside the radiation field (D). CT scans taken on day 14 (E), day 33 (F), day 49 (G), and day 82 (H) of readmission showing development of the cavitary lesion.

A 68‐year‐old male patient with squamous cell carcinoma (cT4N2M0) of the left upper lobe was admitted to our hospital for the introduction of CRT. He had no notable medical history but smoking history of 36 pack‐years. The computed tomography (CT) scan showed a hilar tumour causing atelectasis of the left upper lobe (Fig. 1A). He received CRT consisting of radiotherapy (54 Gy/27 Fx) and concurrent nanoparticle albumin‐bound paclitaxel (100 mg/m^2^) and carboplatin (area under the curve, AUC = 4) on days 1, 15, and 29. In spite of mild oesophagitis, he completed CRT as scheduled and the CT scan revealed marked decrease in the primary lesion and resolution of the atelectasis (Fig. 1B). Then, he received the first dose of durvalumab (10 mg/kg, every two weeks). Nine days after the second dose, he visited our outpatient clinic complaining of fever. The CT scan showed lung infiltrate in the left upper lobe, which was outside the radiation field (Fig. 1C, D). As irAE or bacterial pneumonia was considered, he was readmitted and treated with 25 mg (0.5 mg/kg) of prednisolone as well as 2 g of ceftriaxone daily. On day 14 of readmission, the development of a cavitary lesion was noted on the CT scan (Fig. 1E). We performed bronchoscopy and *Aspergillus fumigatus* was isolated from the specimen. Despite intense treatment including voriconazole followed by liposomal amphotericin B, his fever was sustained and the CT scans showed further development of the cavitary lesion (Fig. 1F, G). Because his general condition worsened and the entire left lung was destroyed (Fig. 1H), the patient underwent a left pneumonectomy on day 88 of readmission.

**Figure 2:**
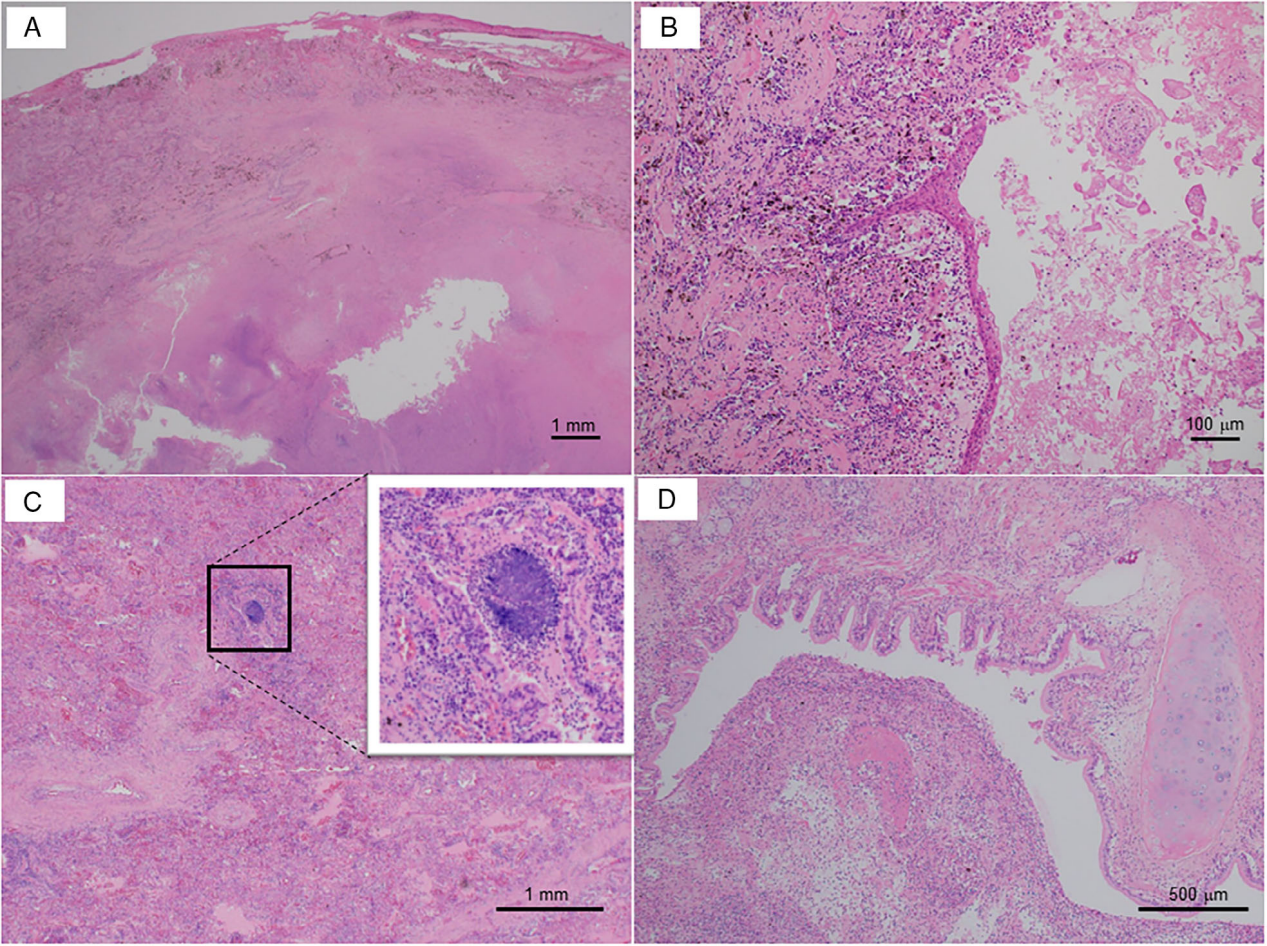
(A–D) The pathology of the removed lung with haematoxylin and eosin stain. (A) A scarred nodule at the site of primary tumour with granulation tissue around (bar = 1 mm). (B) A cavernous lesion having a necrotic substance inside with coagulation necrosis and macrophage infiltration around (bar = 100 μm). (C) Only one colony of *Aspergillus* was identified in the lung tissue (bar = 1 mm). (D) The respiratory tract with organized exudate inside (bar = 500 μm).

The pathology of the removed lung revealed a scarred nodule of 21 mm in diameter at the site of primary tumour with granulation tissue around (Fig. 2A). No cancer cells were found. Separately, a cavernous lesion having a necrotic substance inside was observed, and coagulation necrosis and macrophage infiltration were present around it (Fig. 2B). Only one colony of *Aspergillus* was identified in the lung tissue (Fig. 2C). In the respiratory tract, organized exudate was observed (Fig. 2D).

After surgery, his general condition markedly improved. One year after discharge, he is doing well without any sign of recurrence.

## Discussion

This report has presented a case of destroyed lung in a patient with NSCLC who received CRT followed by durvalumab. Because of the sustained inflammation and abolished function of the left lung, left pneumonectomy was required. In lung pathology, only a scarred nodule with granulation tissue around was observed at the site of primary tumour, indicating that treatment effect of CRT with durvalumab was enough to achieve complete remission of NSCLC. In addition, only one colony of *Aspergillus* was found in the resected lung, suggesting that antifungal treatment also successfully controls the fungal infection. We considered that durvalumab might further accelerate the inflammatory response, which had been introduced by fungal infection, leading to uncontrollable inflammation of the lung.

Immune checkpoint inhibitors are known to enhance host cytotoxic T‐cell immunity, which can lead to dysregulation of the immune system of the host. Cancer immunotherapy is associated with irAEs, which typically involves the skin, lung, and gastrointestinal tract and endocrine system, although there has been little concern about infectious disease. A couple of recent reports indicated that immune checkpoint inhibitors can enhance the immune response to microorganisms and provoke paradoxical reactions [2, 3]. The case described by Uchida et al. had underlying chronic progressive pulmonary aspergillosis that commenced acute progression after 20 cycles of nivolumab [2]. In a case report by Gupta et al., an NSCLC patient with diabetes developed invasive aspergillosis after four cycles of durvalumab following six cycles of chemotherapy with paclitaxel and carboplatin [3]. In contrast, our patient had no underlying disease that could contribute to the development of pulmonary mycosis. In the present case, lung inflammation, possibly in response to *Aspergillus* infection, could be enhanced and sustained by cancer immunotherapy with durvalumab. Even after the fungal infection was controlled, intense lung inflammation prolonged and the function of the left lung was abolished. Therefore, we hypothesized that the present case might be an instance of immune reconstitution inflammatory syndrome.

In this case, although we performed steroid therapy in addition to intense antifungal treatment, progressive tissue destruction could not be broken off. In case of severe but localized inflammation that cannot be controlled by steroid therapy, surgery could be an option.

Immune checkpoint inhibitors theoretically do not cause immunosuppression. However, Fujita et al. reported that infectious diseases were identified in 32 (19.2%) of the 167 patients with NSCLC who received nivolumab [4]. Immunosuppressive treatment for irAEs is one of the risk factors for infections in patients receiving immune checkpoint inhibitors. In addition, immunosuppression associated with immune checkpoint‐related leucopenia/lymphopenia and a hypersensitivity response may also contribute to the risk of infectious diseases [5]. In our patient, the precedent CRT might have induced transient immunosuppression followed by enhanced inflammatory response by durvalumab.

### Disclosure Statement

Appropriate written informed consent was obtained for publication of this case report and accompanying images.
